# A first-in-human study of ^11^C-MTP38, a novel PET ligand for phosphodiesterase 7

**DOI:** 10.1007/s00259-021-05235-0

**Published:** 2021-02-10

**Authors:** Manabu Kubota, Chie Seki, Yasuyuki Kimura, Keisuke Takahata, Hitoshi Shimada, Yuhei Takado, Kiwamu Matsuoka, Kenji Tagai, Yasunori Sano, Yasuharu Yamamoto, Maki Okada, Tatsuya Kikuchi, Masanori Ichise, Kazunori Kawamura, Ming-Rong Zhang, Makoto Higuchi

**Affiliations:** 1 Department of Functional Brain Imaging, National Institute of Radiological Sciences, National Institutes for Quantum and Radiological Science and Technology, 4-9-1 Anagawa, Inage-ku, Chiba, Chiba 263-8555 Japan; 2 Department of Psychiatry, Kyoto University Graduate School of Medicine, 54 Shogoin Kawahara-cho, Sakyo-ku, Kyoto, Japan; 3 Department of Clinical and Experimental Neuroimaging, Center for Development of Advanced Medicine for Dementia, National Center for Geriatrics and Gerontology, 7-430 Morioka, Obu, Aichi Japan; 4 Department of Neuropsychiatry, Keio University School of Medicine, 35 Shinanomachi, Shinjuku, Tokyo, Japan; 5 Department of Psychiatry, The Jikei University Graduate School of Medicine, Tokyo, 105-8461 Japan; 6 Department of Radiopharmaceuticals Development, National Institute of Radiological Sciences, National Institutes for Quantum and Radiological Science and Technology, 4-9-1 Anagawa, Inage, Chiba, Chiba Japan

**Keywords:** ^11^C-MTP38, PDE7, Positron emission tomography, Quantification

## Abstract

**Purpose:**

Phosphodiesterase 7 (PDE7) is an enzyme that selectively hydrolyses cyclic adenosine monophosphate, and its dysfunction is implicated in neuropsychiatric diseases. However, in vivo visualization of PDE7 in human brains has hitherto not been possible. Using the novel PET ligand ^11^C-MTP38, which we recently developed, we aimed to image and quantify PDE7 in living human brains.

**Methods:**

Seven healthy males underwent a 90-min PET scan after injection of ^11^C-MTP38. We performed arterial blood sampling and metabolite analysis of plasma in six subjects to obtain a metabolite-corrected input function. Regional total distribution volumes (*V*_T_s) were estimated using compartment models, and Logan plot and Ichise multilinear analysis (MA1). We further quantified the specific radioligand binding using the original multilinear reference tissue model (MRTM_O_) and standardized uptake value ratio (SUVR) method with the cerebellar cortex as reference.

**Results:**

PET images with ^11^C-MTP38 showed relatively high retentions in several brain regions, including in the striatum, globus pallidus, and thalamus, as well as fast washout from the cerebellar cortex, in agreement with the known distribution of PDE7. *V*_T_ values were robustly estimated by two-tissue compartment model analysis (mean *V*_T_ = 4.2 for the pallidum), Logan plot, and MA1, all in excellent agreement with each other, suggesting the reversibility of ^11^C-MTP38 binding. Furthermore, there were good agreements between binding values estimated by indirect method and those estimated by both MRTM_O_ and SUVR, indicating that these methods could be useful for reliable quantification of PDE7. Because MRTM_O_ and SUVR do not require arterial blood sampling, they are the most practical for the clinical use of ^11^C-MTP38-PET.

**Conclusion:**

We have provided the first demonstration of PET visualization of PDE7 in human brains. ^11^C-MTP38 is a promising novel PET ligand for the quantitative investigation of central PDE7.

**Supplementary Information:**

The online version contains supplementary material available at 10.1007/s00259-021-05235-0.

## Introduction

Cyclic nucleotide phosphodiesterases (PDEs) are 11 genetically related families of phosphohydrolases (PDE1-PDE11). PDEs hydrolyse the second messengers, cyclic adenosine monophosphate (cAMP) and cyclic guanosine monophosphate (cGMP) [1–3], and play crucial roles in regulating various cellular functions.

Among PDEs, the PDE7 family selectively hydrolyses cAMP, and comprises two genes, PDE7A and PDE7B. In rodents and humans, PDE7A is widely expressed in the brain and peripheral organs, while PDE7B is primarily distributed in the brain, including the striatum, thalamus, and hippocampus [4–7]. Pharmacological studies have shown that inhibition of PDE7 has neuroprotective and anti-inflammatory effects and has the potential to improve spatial learning and memory abilities [8]. Moreover, several preclinical studies have suggested the significance of PDE7 as a novel therapeutic target for neuropsychiatric diseases, including Parkinson’s disease [9], Alzheimer’s disease [10], and addiction [11]. In fact, administration of a PDE7 inhibitor induced proliferation of neural stem cells and enhanced dopaminergic neurogenesis in a rat model of Parkinson’s disease [9]. Another study reported that inhibition of PDE7 reversed memory impairments caused by amyloid-beta depositions in the hippocampus and neocortex, and improved hippocampal neurogenesis in a mouse model of Alzheimer’s disease [10]. Therefore, developing methods for visualizing and quantifying PDE7 in human brain would be helpful for etiological assays and therapeutic evaluations of these conditions.

So far, in vivo positron emission tomography (PET) imaging of PDE7 in the brain has not been successful. One research group has documented PET imaging of the mouse brain using PDE7 radioligands (^18^F-MICA-003, ^11^C-MICA-005) [12, 13]. However, it has been concluded that neither of these radioligands is suitable for accurate quantification of central PDE7 because of the emergence of their radiolabeled metabolites in the brain.

We recently developed ^11^C-MTP38 (8-amino-3-(2S*,5R*-dimethyl-1-piperidyl)-[1,2,4]triazolo[4,3-a]pyrazine-^11^C-5-carbonitrile), a radiolabeled compound that has high affinity and selectivity for PDE7. MTP38 acts on PDE7A and PDE7B with half-maximal inhibitory concentration (IC_50_) values of 9.81 nM and 1.21 nM, respectively, and thus is more selective for PDE7B than for PDE7A [14]. In addition, it is much less reactive with other PDEs with IC_50_ exceeding 100 nM [14]. In rat and monkey PET measurements, pre-administration of unlabeled MTP38 blocked radioactivity uptake in the brain, supporting the saturable binding of ^11^C-MTP38 [14]. ^11^C-MTP38 also showed favorable kinetics in these animals for PET quantification of PDE7. Moreover, ^11^C-MTP38 autoradiography demonstrated its specific binding to rodent and non-human primate striatal sections [14]. These results suggest that ^11^C-MTP38 potentially allows in vivo imaging of PDE7 in the human brain.

The purpose of this study was to perform a first-in-human PET imaging of PDE7 in the brains of living subjects with ^11^C-MTP38. We investigated the kinetics of ^11^C-MTP38 by acquiring PET data with arterial blood sampling and metabolite analysis, followed by applications of compartment model, graphical, reference tissue, and standardized uptake value ratio (SUVR) model analyses to the quantitative assessment of radioligand distributions and retentions.

## Materials and methods

### Participants

Seven healthy male volunteers (age, 27.4 ± 7.2 years; body weight, 68.3 ± 8.8 kg; values when applicable are expressed as mean ± SD hereafter) were included in this study. Subjects with current or past psychiatric disorders, substance abuse, current smoking, or organic brain disease were excluded based on their medical history and magnetic resonance imaging (MRI) findings. Subjects also underwent a physical examination and blood and urine analyses to exclude physical illnesses.

This study was approved by the Radiation Drug Safety Committee, and the Institutional Review Board of National Institutes for Quantum and Radiological Science and Technology, Chiba, Japan, and was carried out in accordance with the ethical standards laid down in the 1964 Declaration of Helsinki and its later amendments. After complete description of the study, written informed consent was obtained from all participants. The current study was registered with the Japan Registry of Clinical Trials (jRCTs031190054).

### PET scan procedures

Radiosynthesis of ^11^C-MTP38 was carried out as described previously [14] (Supplementary Fig. 1). All PET scans were conducted with a Biograph mCT flow system (Siemens Healthcare, Erlangen, Germany), which provides 109 sections with an axial field of view (FOV) of 21.8 cm. The intrinsic spatial resolution of this device was 5.9 mm in-plane and 5.5 mm full-width at half-maximum (FWHM) axially. CT scan was performed prior to the emission scan for attenuation correction. Immediately after intravenous rapid bolus injection of ^11^C-MTP38 (injected dose, 349.2 ± 60.1 MBq; molar activities, 14.2 ± 6.8 GBq/μmol; mass, 7.9 ± 3.3 μg), three-dimensional list-mode emission data acquisition was started on a PET camera for 90 min. List-mode data were sorted and rebinned into sinograms with 33 frames of increasing duration from 10 s to 5 min (10 s × 6, 20 s × 3, 1 min × 6, 3 min × 4, and 5 min × 14). Signograms were reconstructed using a filtered back-projection algorithm with a Hanning filter (4.0 mm FWHM). All PET images were corrected for attenuation based on the CT images, for randoms using the delayed coincidence counting method, and for scatter using the single-scatter simulation method. A head fixation device was used to minimize the subject’s head movement during the PET measurements.

To evaluate the safety of ^11^C-MTP38, blood tests including a complete blood cell count and serum biochemistry were conducted before and 90 min after injection of ^11^C-MTP38.

### Measurement of ^11^C-MTP38 in plasma

To obtain individual input functions to be used for the PET scan data analysis, arterial blood samples were taken manually 32 times after radioligand injection at 10-s intervals up to 120 s, 30-s intervals up to 3 min, 1-min intervals up to 10 min, and single 12-, 15-, 20-, 25-, 30-, and 10-min intervals up to 90 min after injection. Arterial blood sampling could not be technically performed in one subject. An aliquot of each blood sample was centrifuged to obtain plasma. Plasma and whole blood radioactivity concentrations were measured with an auto-gamma counter (WIZARD 1480, PerkinElmer, Waltham, MA). The plasma-free fraction was measured by ultrafiltration (Centrifree, Merck Millipore, Billerica, MA) in triplicate. The fractions of the parent and its radiometabolites in plasma were determined by HPLC from six samples in each subject (at 3, 10, 20, 30, 60, and 90 min). A plasma sample was deproteinized by adding an equivalent volume of acetonitrile, and an aliquot of the supernatant obtained by centrifugation was analyzed by radio-HPLC with a semi-preparative column (Capcell Pak C18 AQ, 5 μm, 10 × 250 mm connected with a guard column, Capcell Pak C18 AQ, 5 μm, 10 × 20 mm, Osaka Soda Co., Ltd., Osaka, Japan). An aqueous solution of acetonitrile (50%) was used as mobile phase at a flow rate of 4 mL/min.

### MRI scan procedures

Structural T1-weighted images were acquired for all subjects with a 3-T MRI scanner (MAGNETOM Verio, Siemens, Germany). 3D volumetric acquisition of a T1-weighted gradient-echo sequence produced a gapless series of thin sagittal sections (TE/TR, 1.95/2300 ms; TI, 900 ms; flip angle, 9°; FOV, 250 mm; acquisition matrix, 256 × 256; slice thickness, 1 mm).

### Brain image data processing

Head movement during the PET scan in each subject was corrected by registering all emission frames to the average image of the first 10 frames, with rigid transformation. Two authors (MK and CS) independently performed visual inspection of all PET images and confirmed that there was no apparent intra-frame or inter-frame motion, as well as no mis-registration between the CT and emission images in each subject. The motion-corrected PET images were co-registered to the corresponding individual T1-weighted MR images. For each T1-weighted image, surface-based cortical reconstruction and volumetric subcortical segmentation were performed with FreeSurfer tools (version 6.0.0; http://surfer.nmr.harvard.edu), and the following regions of interest (ROIs) were defined using its atlases [15–17]: frontal, temporal, parietal, occipital, anterior cingulate, posterior cingulate, and insular cortices, thalamus, caudate, putamen, globus pallidus, amygdala, hippocampus, cerebellar cortex, and pons. Motion correction, visual inspection of images, co-registration of PET images into MR images, and kinetic analyses were performed using PMOD® software ver. 3.8 (PMOD Technologies Ltd., Zurich, Switzerland).

### Kinetic analyses of radioligand in the brain

Regional total distribution volume (*V*_T_), a sum of non-displaceable (*V*_ND_) and specific binding (V_S_) distribution volumes, being equal to the tissue-to-plasma ratio of the radioligand concentration at equilibrium, was calculated with compartment models and graphical analyses using arterial input functions. Brain tissue and blood data from six subjects were used for the initial modeling evaluation. For compartment analyses, *V*_T_ was determined with one- (1TCM) and two-tissue compartment (2TCM) models. For graphical analyses, *V*_T_ was estimated by plasma input Logan plot [18] and Ichise multilinear analysis (MA1) [19]. For all kinetic analyses using input functions, the cerebral blood volume contribution to tissue radioactivity was fixed at 5%.

To investigate the minimal scan length required for reliable quantification of *V*_T_, we conducted the analysis by truncating PET data acquisition duration by every 10 min stepwise from 90 min down to 40 min.

### Reference tissue model

To estimate the non-displaceable binding potential (*BP*_ND_) of the radioligand in each brain region, we applied the original multilinear reference tissue model (MRTM_O_) [20] in ROI analysis and parametric imaging, with the latter allowing a voxel-based analysis. MRTM_O_ is known to allow for *BP*_ND_ estimation with the smallest parameter estimation variability compared to other linear (MRTM and MRTM2) or non-linear models. Its limitation is a negative bias in the presence of noise in PET data, and its magnitude increases with noise. However, this negative bias is known to be minimal when the magnitude of *BP*_ND_ is small (< 1) [20].

Reference tissue models use time-activity data in a brain region devoid of specific binding components as input functions instead of arterial data, permitting quantitative measurements in all seven subjects. *BP*_ND_ was defined as

$$ {BP}_{\mathrm{ND}}=\left[{V}_{\mathrm{T}}\left(\mathrm{target}\right)/{V}_{\mathrm{T}}\left(\mathrm{reference}\right)\right]-1, $$

where *V*_T_ (target) and *V*_T_ (reference) are *V*_T_ values of target and reference regions, respectively. We used the cerebellar cortex as a reference region, because only low levels of PDE7 mRNA are reported in the human cerebellar cortex [7]. *BP*_ND_ estimations were performed both with the indirect 2TCM method and with MRTM_O_.

### SUVR method

Finally, we calculated (SUVR-1) to examine the possibility of quantification of ^11^C-MTP38 specific binding with shorter scan length without requirement of arterial blood data. We obtained (SUVR-1) ROI values from the summed PET images for 40–60, 50–70, 60–80, and 70–90 min normalized to the cerebellar cortex.

### Statistical analysis

An optimal compartment model was chosen on the basis of the Akaike information criterion (AIC) [21], model selection criterion (MSC) [22], and goodness of fit assessed with *F* statistics [23]. In a model with better fitting, AIC showed lower values. A *P* value of less than 0.05 was considered significant for the *F* test. The standard error (SE) of kinetic parameters was given by the diagonal of the covariate matrix. Divided by the estimate of the parameter itself, SE was expressed as a percentage and used to assess parameter identifiability. A smaller percentage indicates better identifiability.

Pearson *r* and linear regression analyses were used to assess (1) correlations between *V*_T_ values estimated with an optimal compartment model and those estimated with graphical analyses, (2) correlations between *BP*_ND_ values estimated by indirect kinetic method and those estimated with MRTM_O_ on a ROI basis, (3) correlations between *BP*_ND_ values estimated with MRTM_O_ on a ROI basis and those estimated with MRTM_O_ on a voxel-by-voxel basis, and (4) correlations between *BP*_ND_ values estimated by indirect kinetic method and (SUVR-1) values using PET data from different scan intervals.

For one subject without arterial blood sampling, full kinetic analyses were not performed. The imaging data of this subject was used for investigation of the time-course of radioactivity and for reference tissue model analysis (*BP*_ND_ estimations with MRTMo on (1) a ROI basis and (2) a voxel-by-voxel basis, as well as correlations between *BP*_ND_ estimations by these two methods).

## Results

### Safety

There were no adverse or clinically detectable pharmacologic effects in any of the subjects (*n* = 7). No significant changes in vital signs or the results of laboratory studies were observed.

### Plasma analysis

**Fig. 1:**
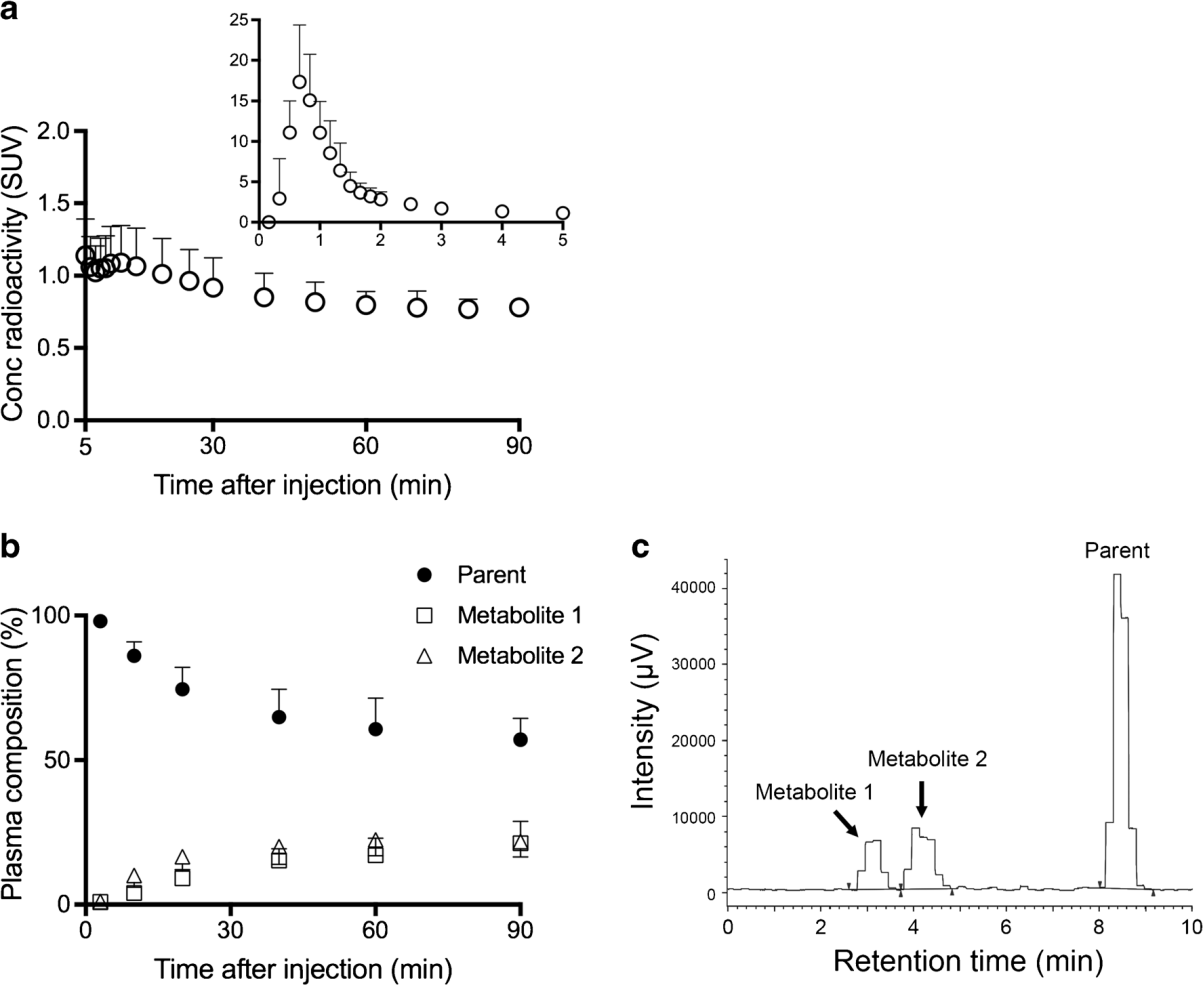
Concentration of radioactivity and composition of plasma activity in arterial plasma after injection of ^11^C-MTP38. **a** Concentration of radioactivity in plasma. Values from 0 to 5 min and 5 to 90 min are shown in the two graphs with different y-axis ranges. **b** Composition of plasma activity. Data points and error bars represent the mean and SD from six subjects. **c** Representative radiochromatogram at 20 min after injection of ^11^C-MTP38

Radioactivity in plasma showed a rapid increase and fast washout after the radioligand administration (Fig. 1a). On average, more than 50% of the radiotracer remained unmetabolized in plasma 90 min after the injection of ^11^C-MTP38 (Fig. 1b). Two radiometabolites of ^11^C-MTP38 appeared in plasma of all six subjects undergoing blood sampling after radioligand injection, and they were more hydrophilic than the parent radioligand as judged by their retention times on reverse-phase HPLC charts (Fig. 1c). The plasma-free fraction of ^11^C-MTP38 was moderate, and was quantified as 23.3 ± 2.2.

### Brain uptake and kinetic analysis

**Fig. 2:**
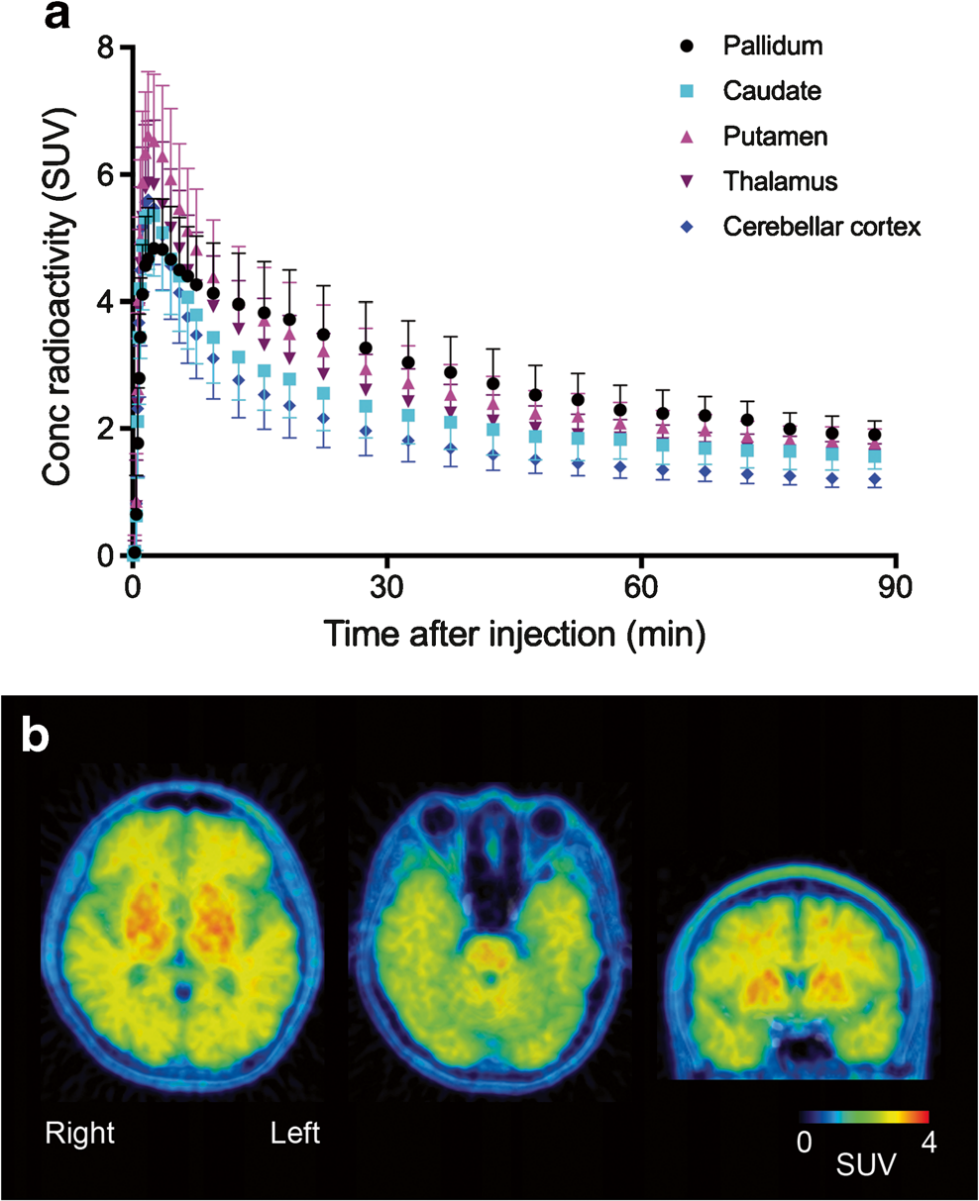
Average time-course of radioactivity and a representative image after injection of ^11^C-MTP38. **a** Average time-course of radioactivity in the pallidum, caudate, putamen, thalamus, and cerebellar cortex. Data points and error bars represent the mean and SD from seven subjects. **b** Representative image from a subject injected with ^11^C-MTP38. PET image was obtained by averaging from 90 min after the injection, and was fused with the corresponding T1-weighted MR image

After injection of ^11^C-MTP38, brain radioactivity peaked within a few minutes with standardized uptake values ranging from 4 to 8, which are sufficiently high for brain imaging. Prompt washout from the cerebellar cortex then took place, while radioactivity washout was relatively slow in the pallidum and putamen where PDE7 is present at high density (Fig. 2).

**Fig. 3:**
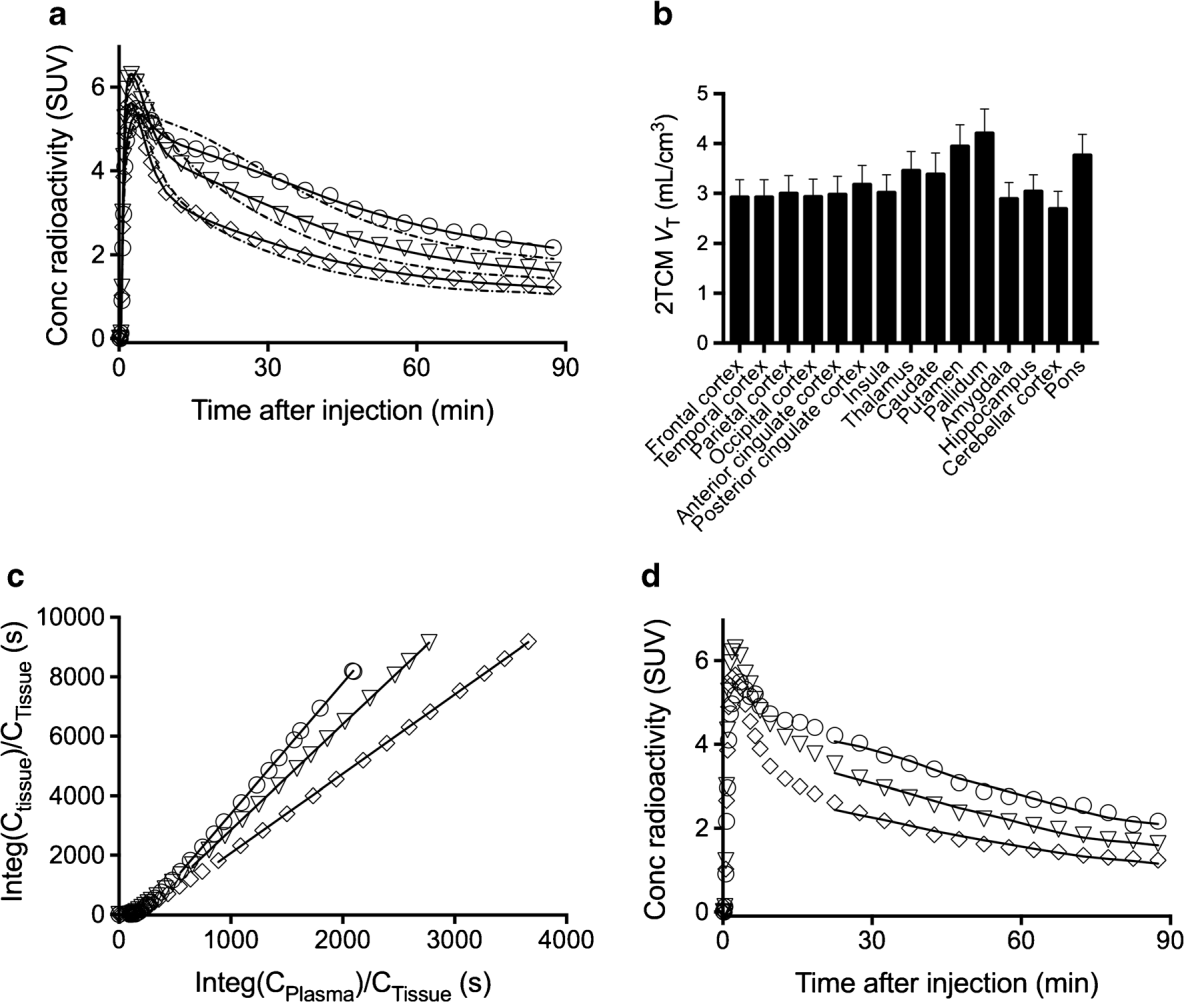
Results of compartment models and graphical analyses. **a** Concentrations of radioactivity in the pallidum (circles), thalamus (triangles), and cerebellar cortex (diamonds) after injection of ^11^C-MTP38 fitted with one-tissue compartment model (1TCM, dotted lines) and two-tissue compartment model (2TCM, solid lines). **b** *V*_T_ values estimated with 2TCM in various regions in six subjects with arterial blood sampling. **c** Curve fitting by Logan plot in the same subject. The plots are linear after 20 min (t*). **d** Curve fitting by MA1 in the same subject after 20 min (t*)

*V*_T_ was robustly estimated for all ROIs by analyzing 90-min PET data and plasma input functions with 2TCM, Logan plot, and MA1. Regional *V*_T_ rank orders were consistent with the known distribution of PDE7 [7]. Mean *V*_T_ values estimated with 2TCM were highest in the pallidum (4.2) followed by the putamen (3.9), and lowest in the cerebellar cortex (2.7). For compartment model analysis in the six subjects whose arterial input functions were available, 2TCM described the tissue TACs better than 1TCM (Fig. 3a). AIC, MSC and *F* test indicated that two-tissue fitting (AIC, −35 ± 27; MSC, 5.9 ± 0.9) was superior to one-tissue fitting (AIC, 48 ± 16; MSC 3.4 ± 0.5) across the ROIs (*P* < 0.000001 by *F* test for all regions). The estimation of *V*_T_ by 2TCM was very stable, with mean SE of 1.4%. SE was less than 5% in all regions and subjects, except for the caudate in one subject with fairly stable SE of 7.3% (Table 1, Fig. 3b). The Logan and MA1 graphical plots exhibited linearity beyond an estimated equilibrium time (t*) of 20 min in all regions and subjects (Fig. 3c, d). We investigated whether changing t* between 20 and 60 min would affect the *V*_T_ estimation by these graphical analyses, and we found that changing t* did not markedly alter the *V*_T_ values (Supplementary Fig. 2).

**Table 1:** Regional total distribution volume (*V*_T_) by two-tissue compartment model (2TCM) using parent as input function. *N* = 6 with arterial blood sampling. Values are mean ± SD

	*V*_T_ (mL·cm^−3^)
Region	by 2TCM
Frontal cortex	2.9 ± 0.4
Temporal cortex	2.9 ± 0.3
Parietal cortex	3.0 ± 0.4
Occipital cortex	2.9 ± 0.4
Anterior cingulate cortex	3.0 ± 0.4
Posterior cingulate cortex	3.2 ± 0.4
Insula	3.0 ± 0.4
Thalamus	3.5 ± 0.4
Caudate	3.4 ± 0.4
Putamen	3.9 ± 0.4
Pallidum	4.2 ± 0.5
Amygdala	2.9 ± 0.3
Hippocampus	3.0 ± 0.3
Cerebellar cortex	2.7 ± 0.3
Pons	3.8 ± 0.4

*V*_T_ values determined with the Logan plot and MA1 were closely correlated with those estimated with 2TCM (*r*^2^ = 0.998 for both models; regression slopes, *Y* = 0.95*X* + 0.07 and *Y* = 0.95*X* + 0.06 for the Logan plot and MA1, respectively; Supplementary Fig. 3). The inter-subject coefficient of variance for *V*_T_ values was less than 14% for all models and regions.

2TCM was also capable of stably estimating *V*_T_ values with truncated dynamic data for all regions. *V*_T_ values determined by 2TCM did not overtly fluctuate by truncating the data from 90 min to 60 min (Supplementary Fig. 4). Meanwhile, the use of dynamic data shorter than 60 min induced large variability of *V*_T_ estimates. The mean identifiability of *V*_T_ values was excellent for analyses with 60-min to 80-min data (mean %SE < 2.3%).

### Quantification of ^11^C-MTP38 binding with MRTM_O_

**Fig. 4:**
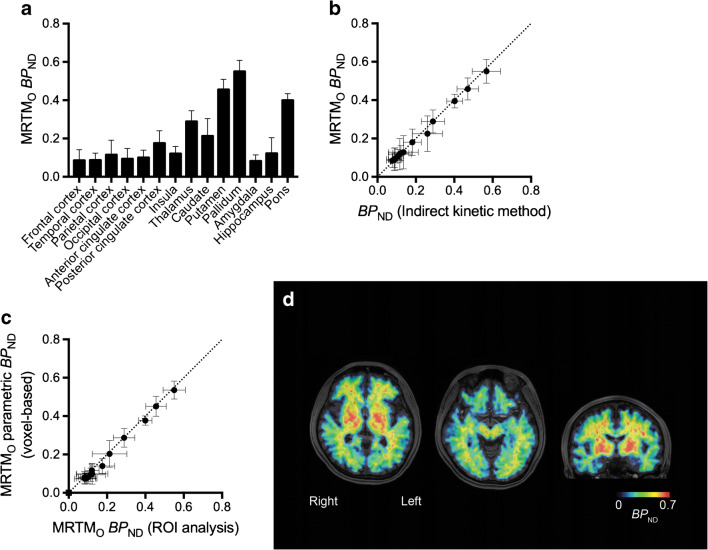
Results of reference tissue model analysis. **a** *BP*_ND_ values estimated by MRTM_O_ in various regions from region of interest (ROI) analysis in seven subjects. **b** Correlation between *BP*_ND_ calculated with MRTM_O_ from ROI analysis and *BP*_ND_ calculated by indirect kinetic method (two-tissue compartment model) using parent as input function in six subjects with arterial blood sampling (*r*^2^ = 1.00). **c** Correlation between *BP*_ND_ calculated with MRTM_O_ from voxel-based parametric images (ROIs placed on parametric images) and corresponding *BP*_ND_ calculated with MRTM_O_ from ROI analysis in seven subjects (*r*^2^ = 0.998). **d** Representative parametric *BP*_ND_ image estimated with MRTM_O_. The PET image was fused with the corresponding T1-weighted MR image. Data points and error bars represent the mean and SD for each region. Straight lines are the lines of identity

*BP*_ND_ values estimated by the MRTM_O_ method were highest in the pallidum (0.55 ± 0.06), followed by the putamen (0.46 ± 0.05) (Table 2, Fig. 4a). *BP*_ND_ values estimated by MRTM_O_ were well correlated with *BP*_ND_ values calculated by the indirect kinetic method with 2TCM (*r*^2^ = 0.997; regression slope, *Y* = 0.96*X* + 0.004; Fig. 4b). In addition, *BP*_ND_ values estimated with MRTM_O_ on a voxel-by-voxel basis using the cerebellar cortical ROI TAC as reference tissue were well correlated with those estimated with ROI-based analysis (*r*^2^ = 0.996; regression slope, *Y* = 0.96*X* − 0.02; Fig. 4c, d).

**Table 2:** Regional binding potential (*BP*_ND_) by indirect kinetic method (2TCM) using parent as input function and by original multilinear reference tissue model (MRTM_O_) with cerebellar cortex as reference. *N* = 6 with arterial blood sampling. Values are mean ± SD

Region	*BP*_ND_	
	Indirect kinetic	MRTM_O_
Frontal cortex	0.09 ± 0.04	0.09 ± 0.06
Temporal cortex	0.09 ± 0.03	0.09 ± 0.04
Parietal cortex	0.12 ± 0.06	0.12 ± 0.08
Occipital cortex	0.09 ± 0.04	0.09 ± 0.06
Anterior cingulate cortex	0.11 ± 0.03	0.11 ± 0.04
Posterior cingulate cortex	0.18 ± 0.05	0.18 ± 0.07
Insula	0.12 ± 0.04	0.12 ± 0.04
Thalamus	0.29 ± 0.06	0.29 ± 0.06
Caudate	0.26 ± 0.08	0.22 ± 0.09
Putamen	0.47 ± 0.06	0.46 ± 0.06
Pallidum	0.57 ± 0.07	0.55 ± 0.06
Amygdala	0.08 ± 0.03	0.08 ± 0.03
Hippocampus	0.14 ± 0.08	0.13 ± 0.09
Pons	0.40 ± 0.04	0.40 ± 0.04

### Quantification of ^11^C-MTP38 binding by SUVR method

The (SUVR-1) values using the summed PET images for 40–60, 50–70, 60–80, and 70–90 min were all well correlated with the *BP*_ND_ values calculated by the indirect kinetic method with 2TCM (*r*^2^ = 0.98–0.99) (Supplementary Fig. 5). The regression slopes of (SUVR-1) values against *BP*_ND_ values were *Y* = 1.20*X* + 0.005, *Y* = 1.18*X* + 0.02, *Y* = 1.12*X* + 0.03, and *Y* = 1.07*X* + 0.03 for the data with time points of 40–60, 50–70, 60–80, and 70–90 min, respectively (Supplementary Fig. 5). The Bland-Altman plots showed smaller proportional biases in regions with small *BP*_ND_ values (*BP*_ND_ < 0.2) for PET images at earlier time points, and in regions with relatively high *BP*_ND_ values (*BP*_ND_ > 0.2) for PET images at later time points (Supplementary Fig. 5). Compared to the *BP*_ND_ values, the SUVR-1 values tended to overestimate, and showed slightly higher inter-individual variability (Supplementary Fig. 5, Supplementary Table 1).

## Discussion

Using a novel PET ligand, ^11^C-MTP38, we established methods to visualize and quantify PDE7 densities in the human brain. No adverse effects of the ^11^C-MTP38 injection were observed. ^11^C-MTP38 showed favorable kinetics for quantification with relatively high uptake in regions including the pallidum, striatum, pons, and thalamus, in line with the known distribution of PDE7. *V*_T_ values were robustly estimated, with 2TCM, Logan plot, and MA1, all in good agreement with each other. The specific radioligand binding to PDE7 could also be estimated as *BP*_ND_ values calculated using MRTM_O_, with the cerebellar cortex as a reference.

Plasma analysis indicated that the metabolic conversion of ^11^C-MTP38 is relatively slow (more than 50% on average remained unmetabolized at 90 min), resulting in high uptake of intact radioligand into the brain. Its uptake was high in brain regions, including the pallidum, putamen, caudate, pons, and thalamus, where abundant PDE7 (mostly PDE7B) expression has been reported. In fact, PDE7B is primarily expressed in the brain, with moderate or high levels noted in the striatum, pallidum, thalamus, and pons according to the Allen Human Brain Atlas (http://portal.brain-map.org; probe A_23_P59338). In addition, a previous study of humans reported that PDE7B mRNA expression levels are highest in the caudate and nucleus accumbens, followed by cortical tissues, hippocampus, and thalamus, while they are low in the cerebellum and dorsal root ganglia; unfortunately, they remained undetermined in the pallidum, putamen, and pons [7]. We also found some radioligand uptake in cerebral white matter following ^11^C-MTP38 injection (Fig. 2b). PDE7 expression in cerebral white matter has not been reported in brains of rodents and humans [5, 7]. Furthermore, our previous PET study in monkeys showed that radioactivity retention around white matter following ^11^C-MTP38 injection was not altered by pretreatment with a PDE7 inhibitor [14]. Thus, the radiosignals in white matter are likely to reflect non-specific radioligand binding.

In our compartment model analyses, *V*_T_ was estimated with excellent identification and good inter-subject variability by 2TCM, as compared with 1TCM, supporting the existence of a compartment for specifically bound ligands. In our graphical analyses, *V*_T_ values estimated by Logan plot and MA1 were in good agreement with those by 2TCM. The Logan and MA1 plots were considered linear after t* = 20 min, indicating that the parent components of TACs reach a transient equilibrium and that ^11^C-MTP38 binding is reversible, which would be suitable for clinical and occupancy studies. It is also noteworthy that our time-stability analysis with 2TCM indicated that scan durations of 60 min or longer stably yield *V*_T_ values. This might indicate, if not confirm, that no major metabolites enter the brain.

We chose MRTM_O_ to quantify the binding of ^11^C-MTP38 with a reference tissue model because it offers stable parameter estimations for regions with a relatively low abundance of binding components. The good agreement between *BP*_ND_ values calculated with MRTM_O_ and the indirect 2TCM method suggests that the reference tissue model with the cerebellar cortex as reference could be useful for reliable quantification of PDE7. Moreover, there was an excellent concordance between *BP*_ND_ values determined by ROI- and voxel-based calculations, supporting the application of parametric *BP*_ND_ images to an exhaustive search for regions and subregions showing altered PDE7 levels. Because MRTM_O_ does not require arterial blood sampling, this method is practical for the clinical use of ^11^C-MTP38-PET. On the other hand, because *BP*_ND_ values of this radioligand are small (0.09 to 0.57 by indirect kinetic methods and 0.09 to 0.55 by MRTM_O_), and the inter-subject variability may increase accordingly, a larger sample size will be needed when applying this radioligand to future clinical and occupancy studies.

We also used the SUVR method for the quantification of ^11^C-MTP38 binding. The advantage of this method is the requirement for a short PET scan length without a need for arterial blood sampling. The validity of this method in the current study was supported by the good correlations between *BP*_ND_ values calculated with indirect 2TCM method and SUVR-1 values. On the other hand, the SUVR-1 values had slight overestimation and slightly higher inter-individual variability compared to *BP*_ND_, as well as stronger proportional biases in regions with relatively high *BP*_ND_ values for PET images at earlier time points, and in regions with small *BP*_ND_ values for PET images at later time points. Considering these factors, it would be preferable to use the SUVR-1 values for the 70–90 min data, focusing analysis on regions where *BP*_ND_ is not too small (*BP*_ND_ > 0.2). Indeed, the SUVR-1 values for 70–90 min in these regions (namely, pallidum, putamen, caudate, pons, and thalamus) showed smaller proportional biases (Supplementary Fig. 5), and the average degrees of overestimation against *BP*_ND_ and inter-individual variability were 15.3% and 23.5%, respectively, suggesting that using these data could minimize the abovementioned effects. However, the selection of quantitative measures (*BP*_ND_ or SUVR-1) and regions of interest should be considered in conjunction with multiple factors including study hypothesis, type of patient populations, and sample size.

There are several points to be considered when applying our first-in-human results to future studies. Firstly, although favorable results have been obtained in our previous occupancy study of ^11^C-MTP38 with a PDE7 inhibitor in monkeys [14], no occupancy or test-retest reproducibility studies have been performed in humans. To further evaluate the kinetics of ^11^C-MTP38 in humans, in addition to our current study, such studies also need to be performed. Next, only male subjects were included in the present study. Thus, although no sex differences in PDE7 distribution have been reported in rodents and humans [5, 7], generalization of our results should be done with caution, and further studies that include both sexes should be conducted.

To conclude, our first-in-human PET imaging of PDE7 has demonstrated that ^11^C-MTP38 is a novel, useful PET ligand for quantifying PDE7 density in the brains of living human subjects. The employment of different analytical models supported desirable kinetic and metabolic properties of ^11^C-MTP38 for neuroimaging assays, and the radioligand binding to PDE7 could also be estimated by both a reference tissue model analysis and SUVR method, circumventing arterial blood sampling. The utilization of ^11^C-MTP38 with this handy and practical protocol could be applied to future clinical studies for in vivo investigations of PDE7 in neuropsychiatric conditions.

## Supplementary information

ESM 1 (PDF 750 kb)

## Data Availability

The datasets generated and analyzed during the current study are available from the corresponding author on reasonable request.

## References

[1] Bender AT, Beavo JA (2006). Cyclic nucleotide phosphodiesterases: molecular regulation to clinical use. Pharmacol Rev.

[2] Keravis T, Lugnier C (2012). Cyclic nucleotide phosphodiesterase (PDE) isozymes as targets of the intracellular signalling network: benefits of PDE inhibitors in various diseases and perspectives for future therapeutic developments. Br J Pharmacol.

[3] Azevedo MF, Faucz FR, Bimpaki E, Horvath A, Levy I, de Alexandre RB (2014). Clinical and molecular genetics of the phosphodiesterases (PDEs). Endocr Rev.

[4] Miro X, Perez-Torres S, Palacios JM, Puigdomenech P, Mengod G (2001). Differential distribution of cAMP-specific phosphodiesterase 7A mRNA in rat brain and peripheral organs. Synapse..

[5] Kelly MP, Adamowicz W, Bove S, Hartman AJ, Mariga A, Pathak G (2014). Select 3′,5′-cyclic nucleotide phosphodiesterases exhibit altered expression in the aged rodent brain. Cell Signal.

[6] Reyes-Irisarri E, Perez-Torres S, Mengod G (2005). Neuronal expression of cAMP-specific phosphodiesterase 7B mRNA in the rat brain. Neuroscience..

[7] Lakics V, Karran EH, Boess FG (2010). Quantitative comparison of phosphodiesterase mRNA distribution in human brain and peripheral tissues. Neuropharmacology..

[8] Morales-Garcia JA, Echeverry-Alzate V, Alonso-Gil S, Sanz-SanCristobal M, Lopez-Moreno JA, Gil C (2017). Phosphodiesterase7 inhibition activates adult neurogenesis in hippocampus and subventricular zone in vitro and in vivo. Stem Cells.

[9] Morales-Garcia JA, Alonso-Gil S, Gil C, Martinez A, Santos A, Perez-Castillo A (2015). Phosphodiesterase 7 inhibition induces dopaminergic neurogenesis in hemiparkinsonian rats. Stem Cells Transl Med.

[10] Bartolome F, de la Cueva M, Pascual C, Antequera D, Fernandez T, Gil C (2018). Amyloid beta-induced impairments on mitochondrial dynamics, hippocampal neurogenesis, and memory are restored by phosphodiesterase 7 inhibition. Alzheimers Res Ther.

[11] Ubaldi M, Cannella N, Ciccocioppo R (2016). Emerging targets for addiction neuropharmacology: from mechanisms to therapeutics. Prog Brain Res.

[12] Thomae D, Servaes S, Vazquez N, Wyffels L, Dedeurwaerdere S, Van der Veken P (2015). Synthesis and preclinical evaluation of an ^18^F labeled PDE7 inhibitor for PET neuroimaging. Nucl Med Biol.

[13] Thomae D, Servaes S, Vazquez N, Wyffels L, Dedeurwaerdere S, Van der Veken P, et al. Synthesis and preclinical evaluation of two novel radioligands for PDE7 imaging in the brain. The 21st International Symposium on Radiopharmaceutical Sciences (ISRS 2015), University of Missouri, Columbia, Missouri, USA, May 26–31, 2015. J Labelled Comp Radiopharm. 2015;58:S295.

[14] Obokata N, Seki C, Hirata T, Maeda J, Ishii H, Nagai Y, et al. Synthesis and preclinical evaluation of [^11^C]MTP38 as a novel PET ligand for phosphodiesterase 7 in the brain. bioRxiv. 2020. 10.1101/2020.10.29.354696. 10.1007/s00259-021-05269-4 PMC8426238 33674894

[15] Fischl B, Salat DH, Busa E, Albert M, Dieterich M, Haselgrove C, et al. Whole brain segmentation: automated labeling of neuroanatomical structures in the human brain. Neuron. 2002;33:341–55. 10.1016/s0896-6273(02)00569-x. 10.1016/s0896-6273(02)00569-x 11832223

[16] Klein A, Tourville J (2012). 101 labeled brain images and a consistent human cortical labeling protocol. Front Neurosci.

[17] Iglesias JE, Van Leemput K, Bhatt P, Casillas C, Dutt S, Schuff N (2015). Bayesian segmentation of brainstem structures in MRI. Neuroimage..

[18] Logan J, Fowler JS, Volkow ND, Wolf AP, Dewey SL, Schlyer DJ (1990). Graphical analysis of reversible radioligand binding from time-activity measurements applied to [*N*-^11^C-methyl]-(-)-cocaine PET studies in human subjects. J Cereb Blood Flow Metab.

[19] Ichise M, Toyama H, Innis RB, Carson RE (2002). Strategies to improve neuroreceptor parameter estimation by linear regression analysis. J Cereb Blood Flow Metab.

[20] Ichise M, Liow JS, Lu JQ, Takano A, Model K, Toyama H (2003). Linearized reference tissue parametric imaging methods: application to [^11^C]DASB positron emission tomography studies of the serotonin transporter in human brain. J Cereb Blood Flow Metab.

[21] Akaike H (1974). A new look at the statistical model identification. IEEE Trans Automat Contr.

[22] Fujita M, Seibyl JP, Verhoeff NP, Ichise M, Baldwin RM, Zoghbi SS (1999). Kinetic and equilibrium analyses of [^123^I]epidepride binding to striatal and extrastriatal dopamine D_2_ receptors. Synapse..

[23] Hawkins RA, Phelps ME, Huang SC (1986). Effects of temporal sampling, glucose metabolic rates, and disruptions of the blood-brain barrier on the FDG model with and without a vascular compartment: studies in human brain tumors with PET. J Cereb Blood Flow Metab.

