# Supplementary material for: A first-in-human study of 11C-MTP38, a novel PET ligand for phosphodiesterase 7
Source: Eur J Nucl Med Mol Imaging. 2021 Feb 10;48(9):2846–55. doi: 10.1007/s00259-021-05235-0 (PMC8263543; doi:10.1007/s00259-021-05235-0)
Supplement: Supplementary file 1 — (PDF 750 kb) [file 259_2021_5235_MOESM1_ESM.pdf]

Title page

Title: A First-in-human Study of ^{11}C-MTP38, a Novel PET Ligand for Phosphodiesterase 7

Authors:

Manabu Kubota[1,2], Chie Seki[1], Yasuyuki Kimura[1,3], Keisuke Takahata[1,4], Hitoshi Shimada[1], Yuhei Takado[1], Kiwamu Matsuoka[1], Kenji Tagai[1,5], Yasunori Sano[1,4], Yasuharu Yamamoto[1,4], Maki Okada[6], Tatsuya Kikuchi[6], Masanori Ichise[1,3], Kazunori Kawamura[6], Ming-Rong Zhang[6], Makoto Higuchi[1]

1 Department of Functional Brain Imaging, National Institute of Radiological Sciences, National Institutes for Quantum and Radiological Science and Technology, 4-9-1 Anagawa, Inage, Chiba, Chiba, Japan

2 Department of Psychiatry, Kyoto University Graduate School of Medicine, 54 Shogoin Kawahara-cho, Sakyo-ku Kyoto, Japan

3 Department of Clinical and Experimental Neuroimaging, Center for Development of Advanced Medicine for Dementia, National Center for Geriatrics and Gerontology, 7-430 Morioka, Obu, Aichi, Japan

4 Department of Neuropsychiatry, Keio University School of Medicine, 35 Shinanomachi, Shinjuku, Tokyo, Japan

5 Department of Psychiatry, The Jikei University Graduate School of Medicine, Tokyo 105-8461, Japan

6 Department of Radiopharmaceuticals Development, National Institute of Radiological Sciences, National Institutes for Quantum and Radiological Science and Technology, 4-9-1 Anagawa, Inage, Chiba, Chiba, Japan

Corresponding author:

Manabu Kubota, M.D., Ph.D.

Department of Functional Brain Imaging, National Institute of Radiological Sciences, National Institutes for Quantum and Radiological Science and Technology

ORCID: 0000-0001-9507-1845

Address: 4-9-1 Anagawa, Inage-ku, Chiba, Chiba 263-8555, Japan

Tel. +81-43-206-3251 Fax. +81-43-253-0396

Email: kubota.manabu@qst.go.jp, m_kubota@kuhp.kyoto-u.ac.jp

Supplementary Figures

Supplementary Fig. 1 Radiosynthesis and chemical structure of ^{11}C-MTP38.

(a) ^{11}C-HCN, CuI, DMF, 180°C, 5 min; (b) 7N NH_3 in CH_3OH, 60°C, 1 min, then 100°C, 3 min, under N_2 flow.

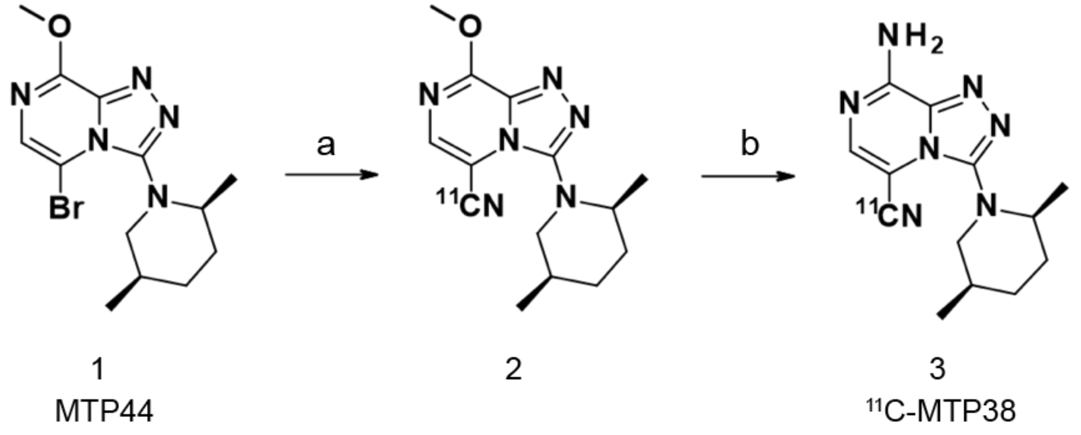

Supplementary Fig. 2 V_T values estimated by (a) Logan plot and (b) MA1 with different equilibrium times (t*). Data points and error bars represent the mean and SD from six subjects with arterial blood sampling.

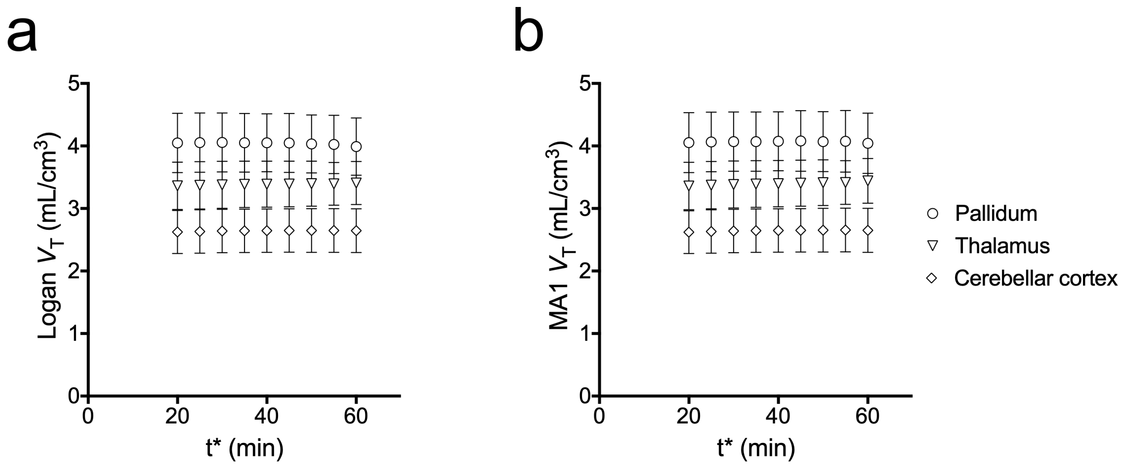

Supplementary Fig. 3 Correlations between V_T values estimated with two-tissue compartment model (2TCM) and by graphical analyses.

The V_T values estimated by (a) Logan plot and (b) MA1 are well correlated with the V_T values estimated with 2TCM ($r^2 = 1.00$ for both). Data points and error bars represent the mean and SD from six subjects for each region. Straight lines are lines of identity.

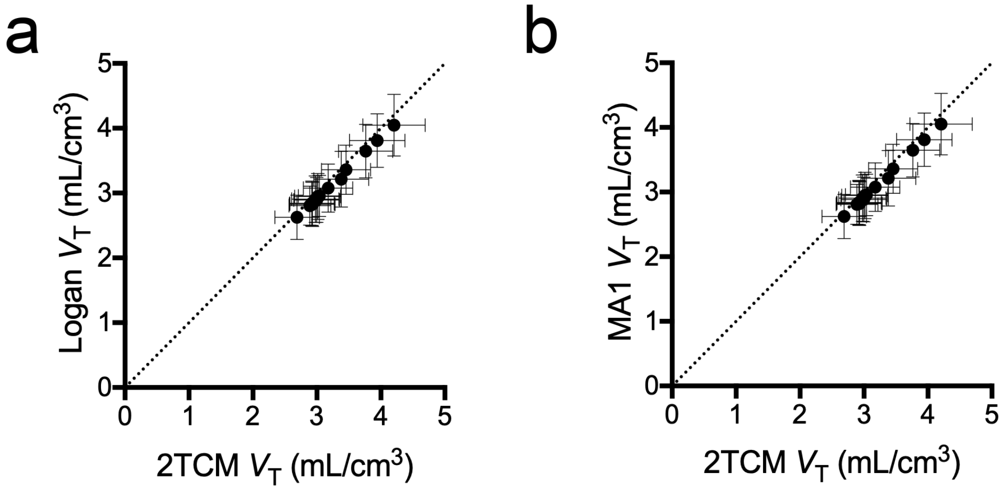

Supplementary Fig. 4 V_T as a function of scan duration. V_T values were estimated with the two-tissue compartment model with scan duration truncated from 90 to 40 min. V_T values are expressed as percentage of the V_T values estimated with the 90-min data. Data points and error bars represent the mean and SD from six subjects with arterial blood sampling. Dotted line indicates 100% of the normalized V_T value.

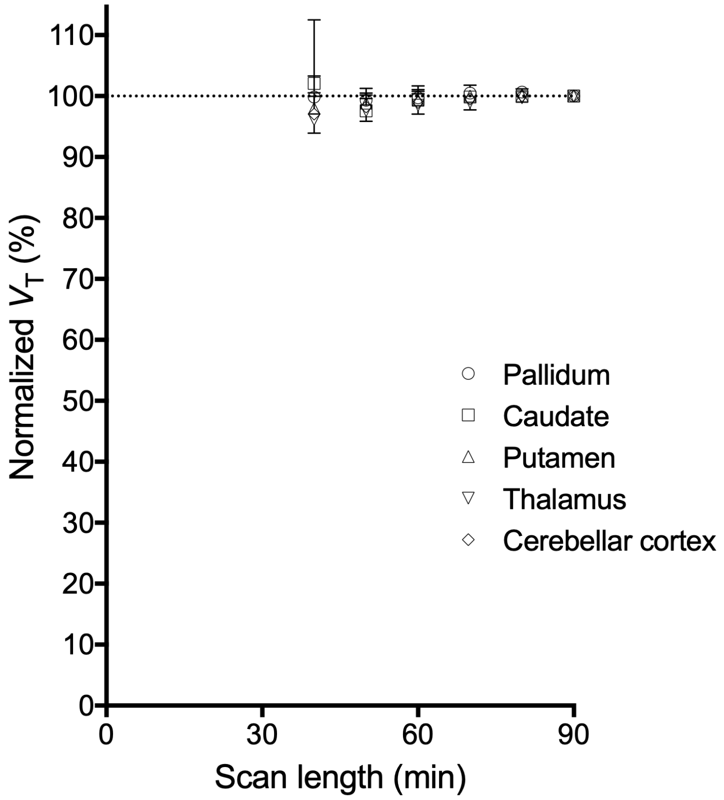

Supplementary Fig. 5 Correlations and Bland-Altman plots between BP_{ND} values calculated with indirect kinetic method (two-tissue compartment model) using parent as input function and (SUVR - 1) values using PET data from different scan intervals (N = 6 with arterial blood sampling). Dotted lines in the Bland-Altman plots indicate 95% confidence intervals.

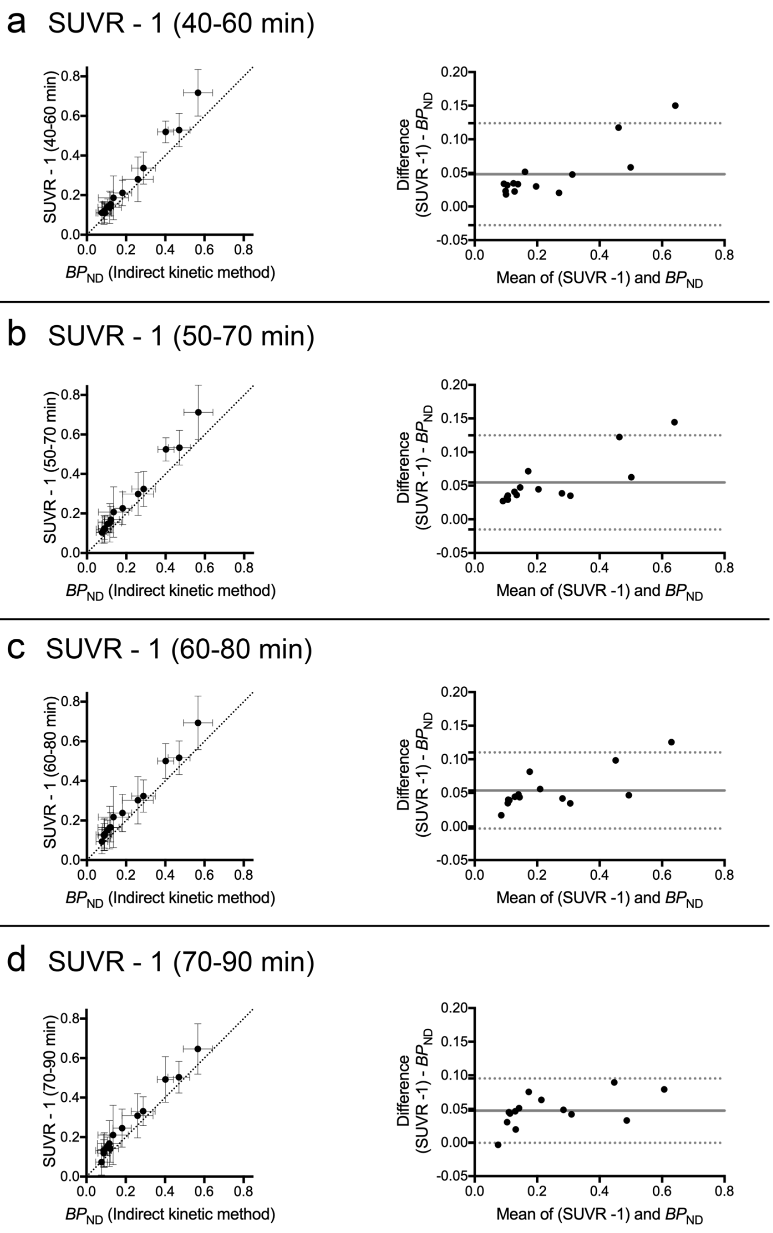

Supplementary Table

Supplementary Table 1 Percent differences between regional (SUVR – 1) values using PET data from different scan intervals and binding potential (BP_{ND}) by indirect kinetic method (N = 6 with arterial blood sampling).

Values are mean ± SD

	Percent difference of (SUVR - 1) relative to BP_{ND}			
Region	SUVR - 1 (40-60 min)	SUVR - 1 (50-70 min)	SUVR - 1 (60-80 min)	SUVR - 1 (70-90 min)
Frontal cortex	24.0 ± 20.9	31.6 ± 28.8	39.1 ± 28.8	44.2 ± 25.4
Temporal cortex	36.2 ± 7.1	36.9 ± 12.8	34.0 ± 20.0	26.0 ± 26.3
Parietal cortex	14.6 ± 15.1	23.5 ± 20.5	32.3 ± 19.1	34.4 ± 22.5
Occipital cortex	17.9 ± 10.0	25.6 ± 18.1	32.6 ± 27.8	35.9 ± 32.3
Anterior cingulate cortex	32.5 ± 14.4	38.2 ± 19.4	41.9 ± 27.1	44.8 ± 25.5
Posterior cingulate cortex	15.4 ± 10.2	22.2 ± 15.3	27.2 ± 16.2	31.6 ± 16.3
Insula	26.2 ± 7.9	37.3 ± 10.2	33.6 ± 17.3	11.9 ± 14.6

Thalamus	15.5 ± 6.3	10.3 ± 11.5	11.0 ± 8.6	15.2 ± 11.2
Caudate	2.1 ± 28.3	11.4 ± 23.6	11.4 ± 34.8	16.4 ± 29.5
Putamen	11.9 ± 5.6	12.7 ± 6.1	9.4 ± 6.1	6.5 ± 5.6
Pallidum	26.1 ± 8.1	24.8 ± 12.1	21.3 ± 10.1	13.2 ± 9.2
Amygdala	38.5 ± 24.9	31.1 ± 24.0	19.8 ± 46.5	-14.4 ± 46.7
Hippocampus	38.2 ± 10.8	56.0 ± 29.5	54.3 ± 24.9	43.7 ± 44.2
Pons	29.2 ± 3.4	28.3 ± 5.9	24.0 ± 13.5	21.1 ± 19.8
